# Heterochromatic siRNAs and DDM1 Independently Silence Aberrant 5S rDNA Transcripts in *Arabidopsis*

**DOI:** 10.1371/journal.pone.0005932

**Published:** 2009-06-16

**Authors:** Todd Blevins, Olga Pontes, Craig S. Pikaard, Frederick Meins

**Affiliations:** 1 Friedrich Miescher Institute for Biomedical Research, Basel, Switzerland; 2 Department of Biology, Washington University in St. Louis, St. Louis, Missouri, United States of America; Duke University, United States of America

## Abstract

5S ribosomal RNA gene repeats are arranged in heterochromatic arrays (5S rDNA) situated near the centromeres of *Arabidopsis* chromosomes. The chromatin remodeling factor DDM1 is known to maintain 5S rDNA methylation patterns while silencing transcription through 5S rDNA intergenic spacers (IGS). We mapped small-interfering RNAs (siRNA) to a composite 5S rDNA repeat, revealing a high density of siRNAs matching silenced IGS transcripts. IGS transcript repression requires proteins of the heterochromatic siRNA pathway, including RNA polymerase IV (Pol IV), RNA-DEPENDENT RNA POLYMERASE 2 (RDR2) and DICER-LIKE 3 (DCL3). Using molecular and cytogenetic approaches, we show that the DDM1 and siRNA-dependent silencing effects are genetically independent. DDM1 suppresses production of the siRNAs, however, thereby limiting RNA-directed DNA methylation at 5S rDNA repeats. We conclude that DDM1 and siRNA-dependent silencing are overlapping processes that both repress aberrant 5S rDNA transcription and contribute to the heterochromatic state of 5S rDNA arrays.

## Introduction

RNA silencing collectively refers to processes by which specialized RNA-protein complexes repress gene expression, guide repressive covalent modifications to histones or mediate DNA methylation within specific DNA regions [1]–[4]. Short-interfering RNAs (siRNAs) are the ∼20–25 nt guide RNAs that afford sequence-specificity to RNA silencing. Most *Arabidopsis* siRNAs depend on RNA polymerase IV (Pol IV), a plant-specific enzyme composed of subunits homologous to RNA polymerases I, II and III [5]–[10]. DNA methylation of repetitive elements correlates with transcriptional silencing and formation of a heterochromatic state, conditions which are thought to preserve genome integrity in eukaryotes [11]–[15].

In current models, Pol IV generates precursors of “heterochromatic” siRNAs from repeat regions, such as the retroelement, *AtSN1* [16], [17]. Pol IV is speculated to produce single-stranded RNA transcripts that are converted into double-stranded RNA (dsRNA) by RNA-DEPENDENT RNA POLYMERASE 2 (RDR2) and processed into siRNA duplexes by DICER-LIKE 3 (DCL3) [7], [18], [19]. The effector protein AGO4 preferentially binds heterochromatic siRNAs [20], [21], which appear to guide RNA-directed DNA methylation (RdDM) [4]. The cytosine methyltransferase, DRM2, is thought to methylate cytosines targeted for RdDM [22]. A second plant-specific RNA polymerase, Pol V (previously known as Pol IVb), mediates production of non-coding transcripts critical for steps downstream of siRNA biogenesis [8], [9], [19], [23]. Additional proteins have also been implicated in these downstream steps [24], [25].

In addition to their role in silencing transposable elements, Pol IV-dependent siRNAs also arise from essential genes, including 5S rRNA gene repeats [18], [26]. 5S rRNA is a structurally conserved, integral component of the ribosome large subunit in all organisms and is essential for ribosome function [27]–[29]. 5S rRNA genes are arranged in tandem repeat arrays (5S rDNA) situated in pericentromeric regions of *Arabidopsis* chromosomes, totaling approximately 1000 repeat copies [30], [31]. Genic portions of active repeats, located on Chromosomes 4 and 5 in the *A. thaliana* ecotype Col-0, are transcribed by Pol III to produce 120 nucleotide (nt) 5S RNA transcripts [31]–[33]. It is not yet clear what proportion of these 5S genes is active at any one time. Circumstantial evidence indicates that Pol IV may play a role in silencing at 5S rDNA loci. For example, siRNAs matching 5S rDNA sequences associate with AGO4 *in vivo* [5], [21] and 5S rDNA methylation is reduced in mutants deficient for the heterochromatic siRNA pathway, such as *nrpd1* (Pol IV largest subunit), *rdr2*, *dcl3*, *ago4* and *drm2* [6], [7], [18], [19]. Moreover, Pol IV physically colocalizes with 5S gene loci, as well as 45S rRNA gene loci [19]. The consequences of DNA methylation changes remain unclear, however, because steady-state levels of major 5S rRNA species are not affected by reduced 5S rDNA methylation [34].

Studies with deficiency mutants have identified multiple systems that maintain DNA methylation in *Arabidopsis* [35]–[38]. Methylcytosine in a ‘CG’ sequence context (CG methylation) is maintained by the methyltransferase MET1 acting in concert with a SWI2/SNF2-like chromatin remodeling factor, DDM1 [39]–[45]. Maintenance of methylcytosine at ‘CHG’ sites (CHG methylation, where H is A, T or C) depends primarily on the methyltransferase CMT3 [46], [47]. Remaining methylcytosine at ‘CHH’ sites (asymmetric methylation) involves DRM2-dependent *de novo* methylation [37], [48]. DDM1 also contributes to the maintenance of CHG and CHH methylation in a subset of genomic sequences [39], [49]. Mutations in *MET1*, *DDM1* and *CMT3* can release silencing of transposable elements [13], [15], [47], [50], whereas their effects on 5S rDNA are more subtle: they derepress “minor” 5S rRNA species whose sequences diverge from “major” species at only one or two positions [51]–[53]. In addition, atypical long transcripts greater than 120 nt in length are generated at 5S rDNA loci in *met1* and *ddm1* mutants [52], [54]. Thus, CG and CHG methylation are implicated in silencing specific subsets of 5S rRNA genes and for preventing production of aberrant transcripts at 5S rDNA loci [31].

The large-scale organization of heterochromatin in the nucleus depends on DDM1 and MET1; nuclei of *ddm1* and *met1* mutants generally show reductions in heterochromatin content relative to wild type nuclei [51], [54], [55]. In *ddm1* mutants, portions of 5S rDNA arrays become localized outside of their normal positions associated with compact “chromocenters” [51]. Heterochromatic marks such as 5-methylcytosine and Histone 3 Lysine 9 methylation also become dispersed relative to the chromocenters in inbred *met1* lines; this is coincident with a progressive, ectopic increase in asymmetric methylation, which is thought to be an siRNA-directed process [54]. The overaccumulation of 5S rDNA-derived siRNAs in DDM1 and MET1-deficient mutants and 5S rDNA decondensation in Pol IV-deficient mutants suggests a role for siRNAs in 5S rDNA chromatin organization [7], [19], [54], [56]. Although DDM1 and heterochromatic siRNAs both appear to function in 5S rDNA condensation, how their functions are coordinated is not known.

Here we demonstrate that Pol IV and other proteins of the heterochromatic siRNA pathway are required for silencing of aberrantly long 5S rDNA transcripts that extend into intergenic spacers downstream of 5S rRNA genes. Production of siRNAs matching these transcripts depends on proteins of this pathway and is limited by the chromatin-remodeling factor DDM1. Our genetic analyses of these phenomena lead us to propose that DDM1-dependent maintenance of silent chromatin, and Pol IV-dependent RNA silencing are overlapping processes that repress aberrant 5S rDNA transcription and contribute to 5S rDNA heterochromatic organization.

## Results

### Distribution of siRNAs representing the 5S rDNA unit repeat

Heterochromatic siRNAs in *Arabidopsis* are typically 23 to 24 nt long and match both strands of corresponding genetic loci [5], [16], [18], [57]. A known 5S rDNA-related species, siR1003, was first characterized by Xie et al. (2004) and matches specific repeats on Chromosomes 3 and 5 [18]. In the present study, we identified 5S rDNA siRNAs in existing high-throughput sequencing data and mapped these to the ∼500 bp unit repeat.

**Figure 1:**
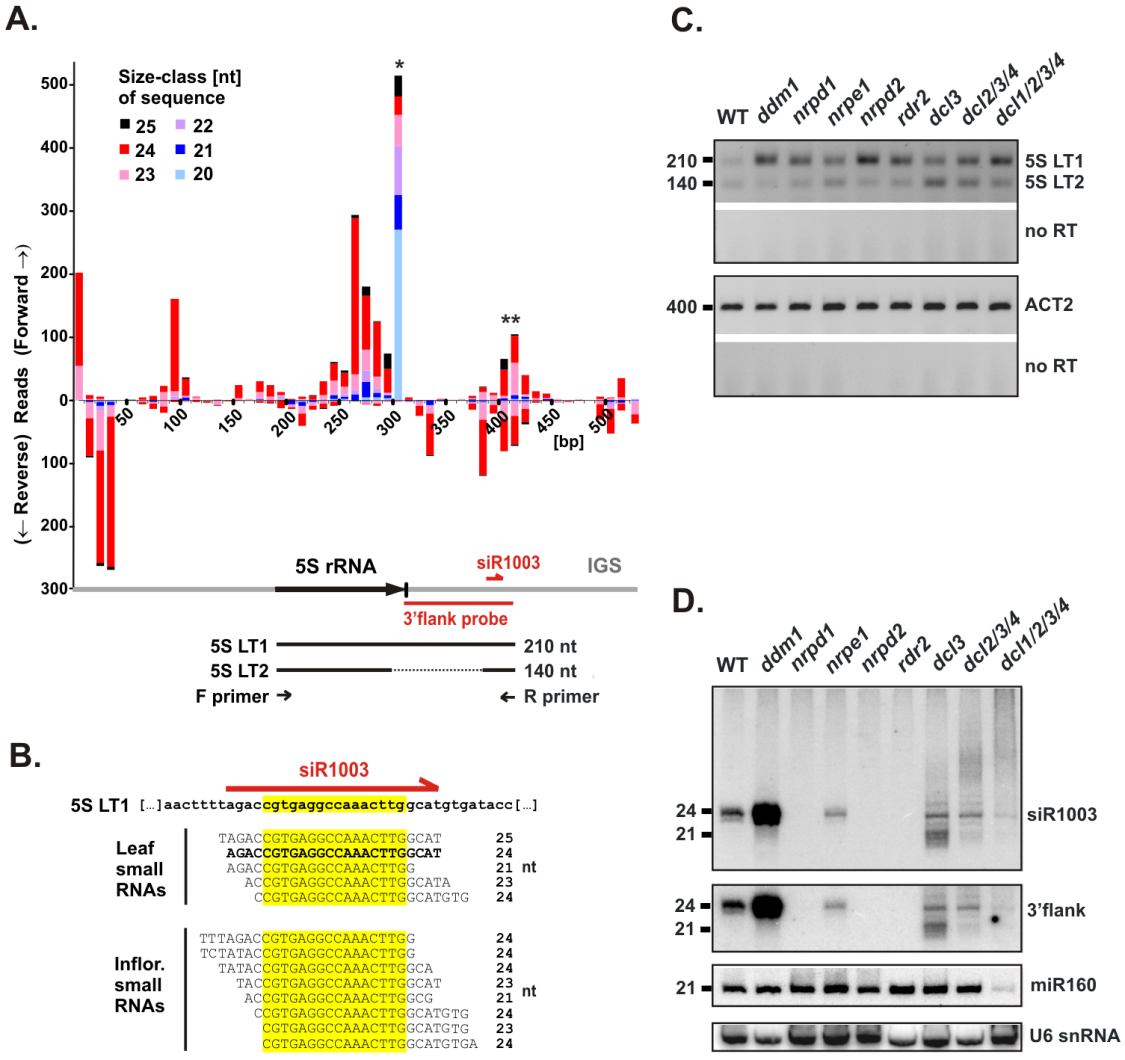
Long 5S rDNA-derived transcripts are subject to RNA silencing. A) Map of *Arabidopsis* small RNAs matching the 5S rDNA unit repeat, based on analysis of leaf datasets from Rajagopalan *et al.* (2006) and Kasschau *et al.* (2007). Small RNA 5′-end positions are indicated on the *x*-axis, with sequencing reads tallied on the *y*-axis. Upward bars are matches to the forward strand; downward bars represent reverse strand matches. Read tallies are stacked in 10-bp bins, with size-class indicated by color. The diagram at bottom shows a 5S rRNA gene (thick black arrow), the surrounding intergenic spacers (IGS, gray), and regions probed by RNA blot hybridization (red lines). RT-PCR products obtained with F and R primers are indicated (black lines). B) Searching small RNA datasets for the siR1003 core sequence (yellow box) yielded a family of 21 to 24-nt siRNAs, which all match 5S LT1 transcripts identified by Vaillant *et al.* (2006) [52]. C) Analysis of 5S LT1 transcript accumulation: RNA samples from inflorescences of wild type (WT), *ddm1* and siRNA biogenesis mutants were analyzed by one-step RT-PCR. Reverse transcription was performed using R primer, and PCR performed using F and R primers (panel A, diagram). Control reactions were performed using ACT2 primers. RT enzyme was omitted from duplicate 5S LT1 and ACT2 reactions (no RT). D) Blot analysis of small RNA isolated from material described in panel C. The membrane was sequentially hybridized with a DNA oligonucleotide (oligo) that detects siR1003, an RNA probe for the 3′-flanking IGS region (panel A, diagram), a DNA oligo that detects miR160, and DNA oligos that detect U6 snRNA. Migration of 21-nt and 24-nt RNA oligo size standards is indicated at left.

Analysis of the origin of siRNAs is complicated by natural variation among the ∼1000 5S rDNA repeats. To resolve this problem, we generated a gapped alignment of 283 individual 5S rDNA repeats derived from an *A. thaliana* YAC library [58]. Next, 5′-end positions of individually matched small RNAs were aligned with a composite 530-bp repeat. **Figure 1A** summarizes our analysis of small RNA datasets from leaves obtained by Rajagopalan *et al.* (2006) and Kasschau *et al.* (2007) [57], [59]. Interestingly, 60% of the 3662 matched sequencing reads represented the intergenic spacer (IGS). A cluster of small RNAs (**Figure 1A**, **) in the IGS matched both strands (44% and 56%, respectively) and included siR1003 (**Figure 1B**). This IGS cluster was also apparent in the maps we generated for other *Arabidopsis* tissues (**Figure S1**, seedlings and inflorescences). The remaining sequences that matched 5S rDNA, 40%, correspond to the 120-bp 5S rRNA genes: these reads were disproportionately 20 to 22-nt long and corresponded predominantly to the 5S rRNA-encoding strand (92% of 1481 genic reads). Analysis of inflorescence data yielded a spike of reads with 5′-ends corresponding to the 5S rRNA 5′-terminus (**Figure S1**). Together with the abundant reads with 3′-ends aligned to the 5S rRNA 3′-terminus (**Figure 1A** and **Figure S1**), this strongly suggests that most genic small RNAs are products of 5S rRNA degradation.

### Pol IV and heterochromatic siRNA production silence 5S rDNA transcripts

Numerous IGS siRNAs occur at positions consistent with the possibility that they derive from aberrant run-on transcripts of 5S rRNA genes that extend into the IGS. To test this hypothesis, we used RT-PCR to measure levels of two long transcripts known to be derived from 5S rDNA, namely: 5S LT1, which is 210 nt in length and extends 90 nt into the 3′ IGS, and 5S LT2, which has the same 5′ and 3′ ends as 5S LT1, but is 70 nt shorter due to an internal deletion ([52], [53], **Figure 1A**).

Mutants homozygous for null alleles of the two largest Pol IV subunits, *nrpd1* and *nrpd2*, showed higher levels of 5S LT1 than wild type plants; these elevated levels were comparable to 5S LT1 derepression in *ddm1* (**Figure 1C**). Other mutants disrupting heterochromatic siRNA biogenesis, namely *rdr2* and *dcl3*, also showed higher 5S LT1 levels. In contrast, a mutant deficient for *trans*-acting siRNA biogenesis [60], *rdr6*, did not show 5S LT1 derepression (**Figure S5**). These data suggest that Pol IV/RDR2-dependent siRNA production, but not RDR6-dependent pathways, contribute to silencing of aberrant transcripts that include 5S rRNA gene sequences (120 bp) but continue at least 90 bp downstream into the IGS. Pol V is functionally and structurally distinct from Pol IV and its largest subunit, NRPE1, is unique [8]–[10], [19]. The *nrpe1* mutant showed modest derepression of 5S LT1. Therefore, both plant-specific RNA polymerases are potentially required for suppressing long 5S rDNA transcripts. We did not detect reproducible changes in 5S LT2, the truncated transcript [52].

### Multiple DCLs mediate silencing of 5S rDNA transcripts

Triple (*dcl2/3/4*) and quadruple (*dcl1/2/3/4*) dicer mutants showed slightly more 5S LT1 accumulation than did *dcl3* alone (**Figure 1C**). Thus, alternate DCLs might mediate 5S LT1 silencing, compensating for DCL3 deficiency. To test this hypothesis, we used RNA blot hybridization to analyze genetic requirements for IGS siRNA biogenesis (**Figure 1D**). We confirmed that siRNAs hybridizing to an siR1003 probe accumulate in wild type but not in *nrpd1*, *nrpd2* or *rdr2* mutants [6], [7], [18]. Comparison to **Figure 1C** suggests that the absence of siR1003 in these mutants correlates with derepression of 5S LT1 transcripts. Accumulation of 24-nt siR1003-related siRNAs was also decreased in *dcl3* mutants, but was accompanied by the appearance of alternate siRNAs in the range 21–23 nt (**Figure 1D**), presumably due to the action of other dicers. Overall, the siR1003 signal in the 21 to 24-nt range in each *dcl* mutant roughly correlated with the extent of 5S LT1 silencing (**Figure 1C**). A longer probe spanning a region of the IGS that includes siR1003 (**Figure 1A**, labeled 3′flank), detected siRNA accumulation patterns identical to those detected using the siR1003 probe (**Figure 1D**).

**Figure 2:**
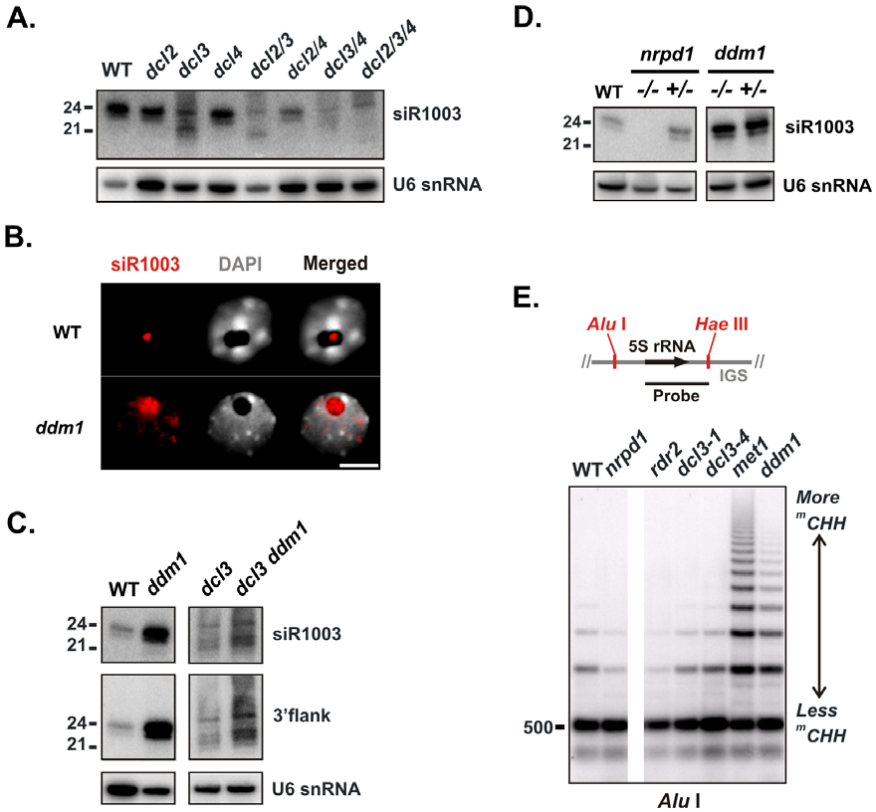
DDM1 limits IGS siRNA accumulation and asymmetric methylation. A) Detection of IGS siRNAs (siR1003) in *dicer-like* (*dcl*) mutant combinations. Blot analysis of small RNA isolated from leaves of wild type (WT); single mutants *dcl2, dcl3, dcl4*; double mutants *dcl2 dcl3* (*dcl2/3*), *dcl2 dcl4* (*dcl2/4*), *dcl3 dcl4* (*dcl3/4*); and the triple mutant *dcl2 dcl3 dcl4* (*dcl2/3/4*). B) Localization of IGS-related RNA in interphase nuclei by fluorescent *in situ* hybridization with an RNA probe for siR1003 (red). DNA was stained with DAPI (white). The size bar corresponds to 5 µm. C) Overaccumulation of IGS siRNAs in the *ddm1* background. Blot analysis of small RNA from *dcl3* and double mutant *dcl3 ddm1* using probes for siR1003 and the larger 3′flanking region (Figure 1A, diagram). WT and *ddm1* signals from the same membrane are provided for comparison. D) IGS siRNA overaccumulation persists in out-crossed *ddm1*. Blot analysis of small RNA isolated from WT, *nrpd1* (−/−), *ddm1* (−/−) and F1 heterozygotes (+/−) of each mutant crossed to the WT. E) Asymmetric cytosines in the IGS are hypermethylated in *met1* and *ddm1*. Southern blot analysis was performed on *Alu* I-digested genomic DNA isolated from WT, *nrpd1*, *rdr2*, two alleles of *dcl3*, *met1* and *ddm1*. The probe corresponds to 5S LT1, shown aligned to a representative 5S rDNA repeat unit from Chromosome 5. *Alu* I and *Hae* III sites in the IGS region are indicated.

To infer processing functions for individual DCLs, we analyzed siR1003 accumulation in double mutant combinations of three DCLs (DCL2, DCL3 and DCL4). We detected ∼22-nt siRNAs in *dcl3*, but this size-class was absent in the double mutant *dcl2/3*. Moreover, ∼21-nt species detected in *dcl2/3* were absent in the triple mutant *dcl2/3/4* (**Figure 2A**). These hierarchical DCL dependencies, previously recognized for other repeat-derived and viral siRNAs [61]–[64], suggest that 21-nt and 22-nt IGS siRNAs are products of DCL4 and DCL2, respectively. Essentially the same results were obtained using probes for the reverse complement of siR1003 (data not shown). Together, our data suggest that dsRNA derived from aberrant 5S rDNA transcripts can be processed by multiple DCLs when DCL3 is mutated, generating IGS siRNAs that contribute to silencing of 5S rDNA.

### DDM1 attenuates IGS siRNA production and RdDM

We confirmed earlier reports that IGS transcript (5S LT1) and siRNA levels are elevated in *ddm1* [7], [52]. Noting this phenomenon, we sought to localize IGS-derived RNA in interphase nuclei using RNA fluorescent *in situ* hybridization (RNA-FISH). In 83% of wild type nuclei (n = 136) the siR1003 probe detected RNA restricted to a distinct, sub-compartment of the nucleolus, a prominent interphase structure devoid of DAPI staining (**Figure 2B**). In contrast, the 5S RNA-FISH signal in 94% of *ddm1* nuclei (n = 158) was typically dispersed throughout the nucleolus and nucleoplasm (see also **Table S1**). These results suggest that IGS-derived siRNAs or their precursors overaccumulate and/or mislocalize in *ddm1* nuclei.

Based on the RNA-FISH result and siRNA overaccumulation observed in both *ddm1* and *met1* mutants [7], [54], we hypothesized that DDM1 and MET1 repress formation of dsRNA precursors that are processed by DCLs to generate IGS siRNAs. If this hypothesis is correct, a double mutant *ddm1 dcl3* should overexpress alternate siRNA size-classes (i.e., those noted in **Figure 2A**). We generated *ddm1 dcl3* by genetic cross and, indeed, detected elevated siRNA levels in *ddm1 dcl3* relative to *dcl3* (**Figure 2C**).

DDM1 and MET1 are required to maintain a silent chromatin state associated with CG methylation [40], [41], [43], which is not immediately re-established upon replacement of functional DDM1 or MET1 [40], [65], [66]. To test whether this silent chromatin state suppresses siRNA precursors, we crossed homozygous *nrpd1* (−/−) and *ddm1* (−/−) lines to wild type Col-0, generating F1 plants that were heterozygous for *nrpd1* (+/−) or *ddm1* (+/−). Re-introduction of a functional *NRPD1* allele in this manner restored siR1003 production to wild-type levels, as expected based on previous complementation and out-cross experiments [5], [19]. In contrast, reintroduction of a functional *DDM1* allele failed to reduce siR1003 overaccumulation (**Figure 2D**). Persistent siRNA overaccumulation and CG hypomethylation in *ddm1* heterozygotes (**Figure S3**) suggests that an epigenetic state correlated with CG methylation, rather than expression of DDM1 *per se*, represses IGS siRNA precursor production.

Next, we tested whether elevated IGS siRNA levels are associated with enhanced RNA-directed DNA methylation (RdDM). Using Southern blot analysis we assayed cytosine methylation in asymmetric sequence contexts (CHH), which largely results from RdDM [35], [67]. After digestion with *Alu* I, which occurs once per 5S gene repeat, 5S rDNA arrays appear on Southern blots as a ladder of bands, each separated by 500-bp intervals. Longer than unit-length (500 bp) fragments reflect *Alu* I site methylation, which is more prominent in *ddm1* and *met1* than in wild type or siRNA biogenesis mutants (*nrpd1*, *rdr2* and *dcl3*) (**Figure 2E**). Methylation is reduced in *nrpd1* and *rdr2* mutants, indicating that siRNA biogenesis is important for *Alu* I site methylation. However, *dcl3* mutants resemble wild-type plants suggesting that other dicers compensate for the loss of 24-nt siRNAs in *dcl3* mutants (see **Figure 2A**). Collectively, the observed increase in DNA methylation in *met1* and *ddm1* mutants is consistent with overproduction of siRNAs leading to increased *de novo* methylation of asymmetric sites. An analogous finding has been reported for siRNA overaccumulation and 5S rDNA hypermethylation in *met1* [54].

### Overlapping Pol IV and DDM1 silencing effects are genetically independent

**Figure 3:**
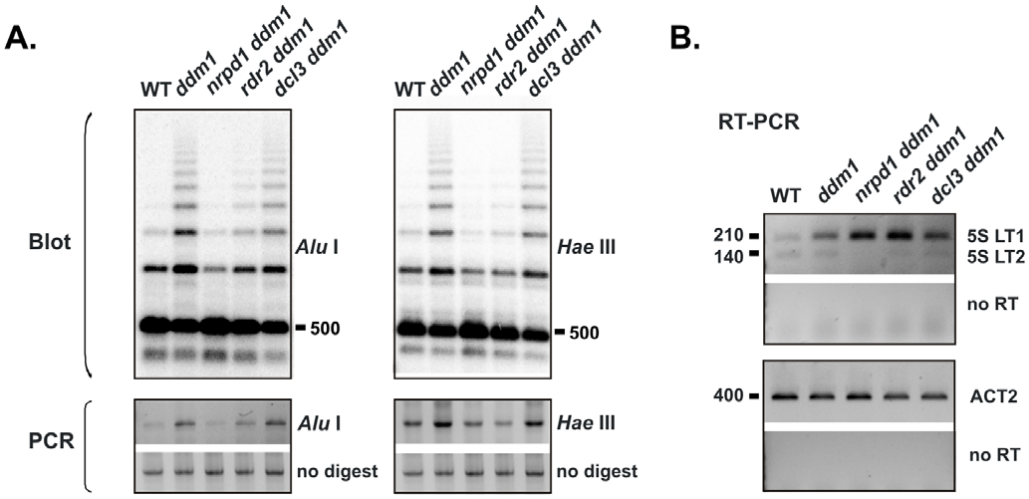
Effect of combined DDM1 and siRNA deficiency on 5S rDNA methylation and aberrant transcripts. A) 5S rDNA hypermethylation in *ddm1* is siRNA-dependent: Southern blot comparison of *Alu*I and *Hae*III-digested genomic DNA isolated from inflorescences of wild type (WT), *ddm1*, and double mutant lines *nrpd1 ddm1*, *rdr2 ddm1*, and *dcl3 ddm1* (top panel). The probe is the same as in Figure 2E. Dilutions of the above digests were also assayed by PCR using 5S LT1 primers (bottom panel). Samples to which no restriction enzyme was added are controls (no digest). B) 5S LT1 is silenced by two overlapping processes: RNA samples from inflorescences of WT, *ddm1* and the double mutant panel were analyzed by one-step RT-PCR, performed as described in Figure 1C. Control reactions were performed with ACT2 primers; reverse transcriptase was omitted from duplicate 5S LT1 and ACT2 reactions (no RT).

To further test whether hypermethylation in *ddm1* is siRNA-dependent, we generated the double mutants *nrpd1 ddm1* and *rdr2 ddm1*, in addition to *dcl3 ddm1* mentioned above. The three double mutants were subjected to Southern blot analysis, alongside wild type and *ddm1* controls. Long *Alu* I fragments typical of *ddm1* mutants were absent in *nrpd1 ddm1* and *rdr2 ddm1* (**Figure 3A**), indicating that *Alu* I site hypermethylation in *ddm1* is indeed siRNA-dependent (i.e., RdDM). *dcl3 ddm1* showed 5S rDNA methylation similar to *ddm1*, again consistent with production of abundant siRNAs of variable lengths in this double mutant (**Figure 2C**). Analysis of *Hae* III methylation, used to test asymmetric 5S rDNA methylation in previous studies [19], [54], yielded identical results (**Figure 3A**). An additional assay using 5S LT1-specific primers, in which PCR amplification indicated DNA methylation status (**Figure 3A**), also supports the hypothesis that the hypermethylation is siRNA-dependent.

Analysis of 5S aberrant transcripts revealed that 5S LT1 accumulates to higher levels in *nrpd1 ddm1* and *rdr2 ddm1* double mutants than in *ddm1* alone; this indicates that both DDM1 maintenance of silent chromatin and siRNA-dependent silencing contribute to suppression of aberrant transcripts (**Figure 3B**). Because siRNA and DDM1 deficiencies are additive in effect, they apparently act in separate pathways.

### Deficiency for DDM1 and siRNAs enhances 5S rDNA decondensation and slows plant growth

**Figure 4:**
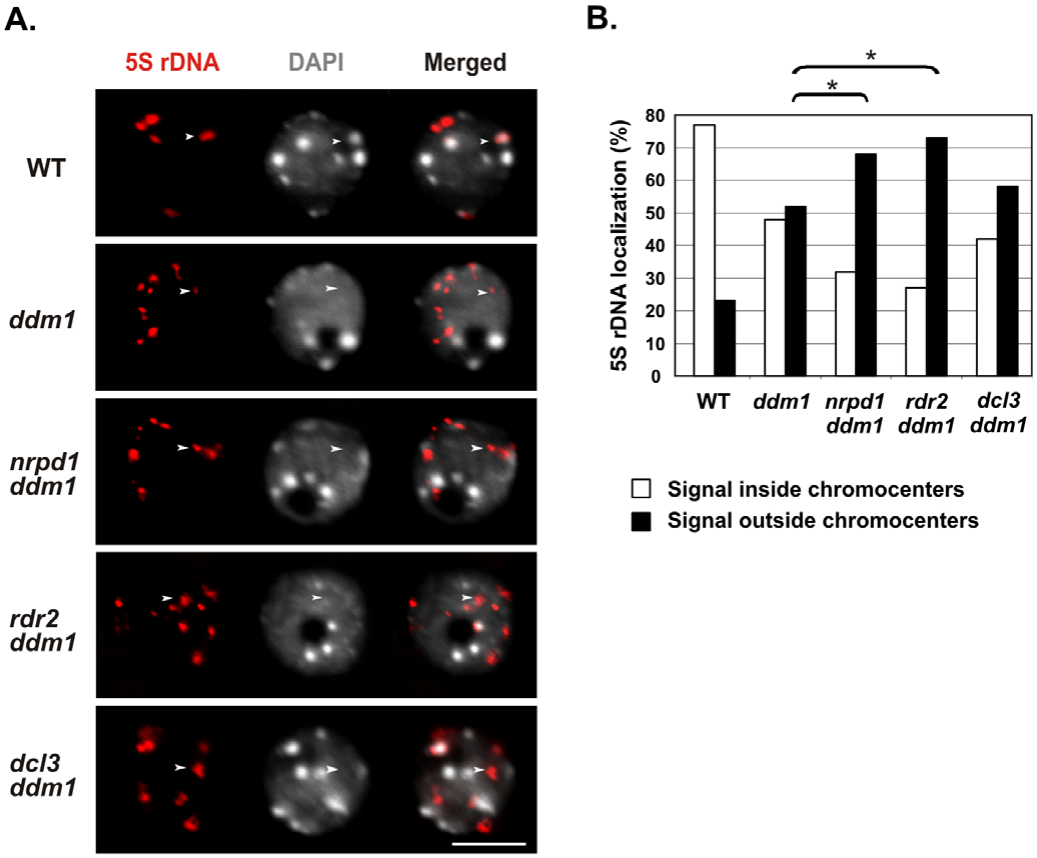
Both DDM1 and siRNAs are required for proper 5S rDNA condensation. A) 5S rDNA localization in interphase nuclei: Fluorescent *in situ* hybridization (red) was performed with a probe for 5S rDNA. DNA was stained with DAPI (white). The size bar corresponds to 5 µm. The white arrow in the wild type (WT) images indicates 5S rDNA colocalized with a DAPI-stained chromocenter; this colocalization was observed in a majority of WT nuclei (see panel B). In contrast, a majority of nuclei from *ddm1* and the double mutants *nrpd1 ddm1*, *rdr2 ddm1* and *dcl3 ddm1* showed 5S rDNA localization outside chromocenters (arrows in mutant panels). B) Nuclei from each genotype were scored for 5S rDNA colocalization with chromocenters (white bars), as compared to 5S rDNA not colocalized with chromocenters (black bars). Differences observed between 5S rDNA localization outside chromocenters in *ddm1* nuclei, compared to in *nrpd1 ddm1* or *rdr2 ddm1* nuclei are statistically significant (*). Numbers of nuclei scored: WT (n = 134), *ddm1* (n = 158), *nrpd1 ddm1* (n = 186), *rdr2 ddm1* (n = 215), *dcl3 ddm1* (n = 190).

The previously observed localization of 5S rDNA outside chromocenters in *ddm1* nuclei implies that 5S rDNA arrays are partially decondensed in *ddm1* [51]. Using DNA-FISH we analyzed nuclei of the *ddm1* mutant in genetic combination with *nrpd1*, *rdr2* or *dcl3*. In the majority of wild type nuclei, 5S rDNA colocalized predominantly with heterochromatic domains known as chromocenters, which stain intensely with DAPI (**Figure 4A**). In contrast, decondensation of 5S rDNA was evident in *ddm1* nuclei, resulting in smaller and more numerous signals. Analysis of 5S rDNA signals colocalized with DAPI-stained chromocenters confirmed that *ddm1* nuclei contained more 5S rDNA outside chromocenters (52%, n = 158) than did wild-type nuclei (23%, n = 134). Importantly, *nrpd1 ddm1* and *rdr2 ddm1* double mutants displayed FISH signal outside chromocenters more frequently (68%, n = 186 and 73%, n = 215, respectively) than did *ddm1*, indicating an additive effect of these mutations on 5S rDNA decondensation (**Figure 4B**). These observed differences were all statistically significant (P<0.05, see **Table S1**). By contrast, 5S rDNA decondensation status in *dcl3 ddm1* (58% outside, n = 190) was not distinguishable from *ddm1*.

**Figure 5:**
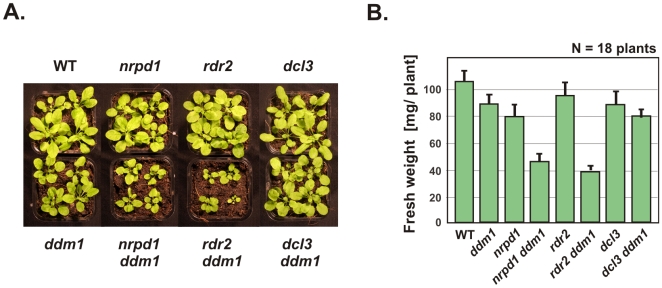
Simultaneous deficiency for DDM1 and siRNAs impairs plant growth. A) Rosette stage plants (21 days-post-germination) of lines deficient for heterochromatic siRNA biogenesis (*nrpd1*, *rdr2* and *dcl3*) compared to corresponding double mutant combinations with *ddm1*. Four representative plants in each population (n = 18) were photographed. B) Fresh weight of aerial portion of plants from each population: plants were individually weighed and mean values plotted with standard errors (bars).

Interestingly, mutants showing pronounced 5S rDNA decondensation also exhibited growth deficiencies; this was particularly evident in the double mutants *nrpd1 ddm1* and *rdr2 ddm1* (**Figure 5A**). The single mutants *nrpd1*, *rdr2*, *dcl3* and *ddm1* showed no appreciable effect on fresh weight (**Figure 5B**). In contrast, *ddm1 nrpd1* and *ddm1 rdr2* double mutant individuals were smaller and weighed, on average, 50% that of *ddm1* or wild-type plants (**Figure 5**). The overall stature and fresh weight of *ddm1 dcl3* plants were similar to those of *ddm1* plants.

## Discussion

Silent chromatin structure is an attribute of tandem repeats in many eukaryotic genomes, including repeats in plants [12], [68]–[70]. *Arabidopsis* 5S rRNA gene loci are interesting in that their cytosine methylation is both DDM1/MET1 and RNA dependent [7], [18], [39], [54]. Earlier findings that DDM1 deficiency perturbs siRNA accumulation suggested that DDM1 might act directly in the RNA silencing pathway [7], [14]. Indeed, our results show that silencing of aberrant 5S rDNA transcripts depends on siRNA biogenesis, in addition to DDM1. However, the contributions of siRNA biogenesis and DDM1 are genetically separable, suggesting that they involve independent mechanisms.

DDM1 limits accumulation of siRNAs derived from the 5S rDNA IGS. Several findings indicate that DDM1 does not directly participate in siRNA production, but helps control transcription of RNAs that serve as precursors for siRNA production. For instance, siRNA overaccumulation persists upon out-cross of *ddm1* mutants to wild-type plants. Moreover, mutants deficient for DDM1, MET1 or VIM methylcytosine-binding proteins gain ectopic (apparently RNA-directed) 5S rDNA methylation but display reduced CG methylation [54], [71]. We therefore posit that a silent chromatin state, maintained by DDM1 and associated with CG methylation, represses production of IGS precursor RNAs that give rise to siRNAs directing DNA methylation. Detection of dsRNA intermediates that are precursors of siRNA duplexes would help test this hypothesis. In addition, analysis of small RNA sequences from *ddm1* (e.g., [49], [72]) could determine whether particular 5S rDNA arrays contribute to siRNA overaccumulation.

**Figure 6:**
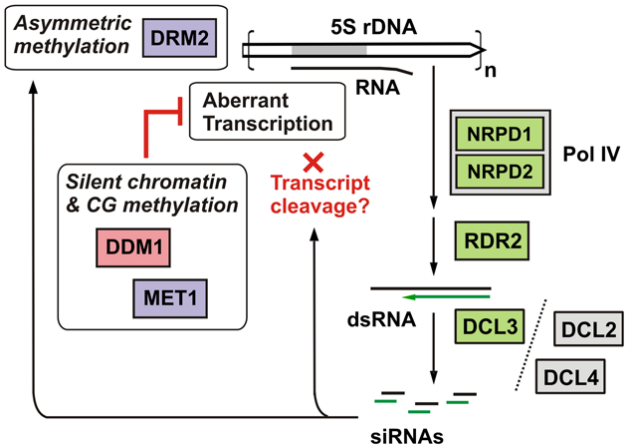
Model for 5S rDNA methylation and aberrant transcript silencing. A canonical RNA polymerase (e.g., Pol III) is assumed to initiate at the 5S rRNA gene promoter, transcribe through the 5S rRNA gene (120 bp, gray), and continue into the intergenic spacer (IGS, white). Concurrently, dsRNA corresponding to the IGS is generated in a Pol IV and RDR2-dependent manner. DCL3 processes dsRNA into 24-nt siRNAs, with DCL2 and DCL4 being alternate enzymes at this step. RNA-directed DNA methylation (RdDM) resulting from this process is thought to require DRM2. DDM1/MET1 maintenance of silent chromatin and CG methylation represses aberrant IGS transcription, limiting production of siRNA precursors and, by consequence, attenuating RdDM. IGS-derived siRNAs may also guide cleavage of nascent IGS transcripts.

In our model (**Figure 6**) a canonical RNA polymerase (e.g., Pol III) is assumed to initiate at the 5S rRNA gene promoter and transcribe into the downstream IGS upon the loss of a silent chromatin state. Pol IV or Pol V transcription can be excluded as the source of these initial transcripts, because 5S LT1 accumulation was not abolished in *nrpd1*, *nrpd2* or *nrpe1* mutants. Concurrently, dsRNA corresponding to the IGS is generated in a Pol IV and RDR2-dependent manner. Multiple DCLs can digest these dsRNA substrates into IGS siRNAs, although DCL3 is overwhelmingly favored. Inhibition of IGS transcription by DDM1 and MET1 would explain why IGS siRNAs overaccumulate in mutants for these proteins. The increased siRNA titers in *ddm1* correlate with hypermethylation of asymmetric CHH sites, and this increased methylation depends on Pol IV and RDR2. DRM2 is thought to mediate such RNA-directed DNA methylation (RdDM) [7], [67], [73], consistent with a report that ectopic 5S rDNA hypermethylation occurring in a *met1* mutant requires DRM2 [54].

Derepression of aberrant 5S rDNA transcripts in *ddm1* is clearly not a total reactivation, because mutants deficient for both DDM1 and siRNA biogenesis showed an additive effect on 5S LT1 derepression. This suggests that siRNA overaccumulation and resulting asymmetric DNA methylation may partially compensate for DDM1 deficiency. However, the enhanced RdDM observed in *ddm1* and *met1* is not sufficient to completely repress 5S LT1 transcripts [52]. Future analysis of 5S LT1 accumulation in mutants deficient for specialized Argonaute effector proteins, such as AGO1 and AGO4, could help address whether RNA silencing of 5S rDNA transcripts occurs by mechanisms other than RdDM.

Beyond 5S rDNA transcript repression, RNA silencing proteins and DDM1 are both required for full condensation of 5S rDNA repeats in the nucleus. Previous studies implicated Pol IV and DDM1 individually in 5S locus condensation [7], [51], [56]. Our study shows that double deficiency for DDM1 and heterochromatic siRNAs causes more decondensation of 5S rDNA than the *ddm1* mutation alone. Furthermore, the reduced biomass of double mutants *nrpd1 ddm1* and *rdr2 ddm1* implies that plant growth responds to both DDM1 and siRNA-dependent signals. This conclusion is consistent with the pronounced phenotype of the double mutant *drm2 met1*, in which both maintenance methylation and RdDM are impaired [54]. The siRNA-dependent pathway has recently been implicated in correcting hypomethylation of transposable element sequences induced by *ddm1* [49]. Taken together, these findings suggest that the balancing of RNA silencing and DDM1/MET1 functions has significance for heterochromatin maintenance, normal growth and development, and protecting the genome from transposon reactivation.

## Materials and Methods

### Mutant plant strains

*nrpd1a-3* (*nrpd1*), *nrpd1b-11* (*nrpe1*), *nrpd2a-1* (*nrpd2*) are SALK T-DNA insertion lines described in Onodera *et al.* (2005) and Pontes *et al.* (2006) [7], [19]. *rdr2-1* and *rdr6-15* are described in Xie *et al.* (2004) and Allen et al. (2005), respectively [18], [74]. *dcl2*, *dcl3* and *dcl4* double and triple mutant combinations are described in Blevins *et al.* (2006) [62], with the constituent alleles being *dcl2-5* of Akbergenov *et al.* (2006) [75], *dcl3-1* of Xie *et al.* (2004) [18] and *dcl4-2* of Xie *et al.* (2005) [76]. *dcl3-4* is a GABI T-DNA insertion described by Daxinger et al. (2008) [77]. *ddm1-2* used for crosses is described in Vongs *et al.* (1993) [39], and *met1-3* in Saze *et al.* (2003) [41].

### Genetic crosses

Quadruple mutant *dcl1/2/3/4* material was obtained by crossing *dcl1-9* (*caf*, [78]) to the triple homozygous mutant *dcl2* (−/−) *dcl3* (−/−) *dcl4* (−/−). F2 progeny of the genotype *dcl1-9*(+/−) *dcl2* (−/−) *dcl3* (−/−) *dcl4* (−/−) were then selected and self-fertilized. About 25% of resulting F3 progeny were quadruple homozygous *d1/2/3/4*. *dcl1-9* used in the above crosses had been introgressed into Col-0 from Ler/Ws backgrounds by 5× outcrossing. Double mutants with *ddm1-2* were generated as follows: *nrpd1*, *rdr2* and *dcl3* were individually crossed to *ddm1*. F2 plants homozygous for both *ddm1* and each RNA silencing mutation were then self-fertilized for two generations and then analyzed with *ddm1* and wild-type lines propagated in parallel.

### Bioinformatic analysis

Sequences of 283 individual 5S rDNA repeats (**File S1**)–from Yeast Artificial Chromosomes (YACs) 4E4, 6A1, 7E7, 7G3, 9A5, 9D3 and 11A3 of the CIC project [58], and representing arrays from chromosomes 3, 4 and 5 [32]–were retrieved from TIGR Plant Repeat Databases [79]. Small RNA datasets of Rajagopalan *et al.* (2006) and Kasschau *et al.* (2007) were retrieved from GEO (http://www.ncbi.nlm.nih.gov/geo) via accession numbers GSM118372 and GSM154370. Sequencing reads from each tissue were combined and filtered to obtain 20–25 nt small RNAs. BLAST (NCBI v2.2.16) was used to query reads to individual 5S rDNA sequences. A Perl script (incorporating Bioperl module Bio::SimpleAlign) converted the 5′-end positions of small RNA hits to individual repeats into coordinates along a multiple alignment of all repeats generated using Clustal W [80] (**File S2**). Tallies of small RNA hits were weighted by number of sequencing reads and collected in 10 nt bins, using the Microsoft Excel 2003 Chart functionality for map generation. Small RNA distribution analyses and maps for leaves, inflorescences and seedlings are presented in **Table S2**, **Figure 1A** and **Figure S2**.

### RT-PCR

RNA was isolated using RNeasy Mini Columns (Qiagen). In brief, 100 mg of mixed-stage inflorescences were ground in liquid nitrogen and processed following the manufacturer's protocol. On-column DNase I digestion was performed (Qiagen), in addition to a second digestion after elution (DNase I Amplification Grade, Invitrogen). Following the protocol of Wierzbicki *et al.* (2008) [23], 400 ng RNA were subjected to gene-specific RT-PCR. To detect 5S LT1 and 5S LT2, primer 5SUNIV2 (R) was used for reverse transcription (SuperScript III, Invitrogen; 50°C for 30 min.). The RT enzyme was then inactivated (70°C for 15 min.), primer RTPCR5S1 (F) added and PCR amplification performed (Platinum Taq, Invitrogen; 52°C annealing, 30 s elongation, 35 cycles). See Vaillant *et al.* (2006) for 5S rDNA primer sequences [52]. ACT2 controls were amplified in separate reactions using primers described in Blevins *et al.* (2006) [62] and different cycling parameters (57°C annealing, 40 s elongation, 25 cycles). Reactions were subjected to agarose gel electrophoresis and stained with ethidium bromide.

### Small RNA blot hybridization

Total RNA was isolated from 600 mg inflorescence tissue by grinding in liquid nitrogen followed by TRIzol extraction (Invitrogen). 100 µg total RNA sample was size-fractionated on RNeasy Mini Columns (Qiagen) as in Blevins *et al.* (2006) [62], then 8 µg low molecular weight RNA was separated on an 18% polyacrylamide gel. Probes for specific RNA species were DNA oligonucleotides end-labeled with γ[^32^P]-ATP: the siR1003 probe described in Xie *et al.* (2004), U6 snRNA probes in Blevins *et al.* (2006) [62], and miR-160 probe 5′-TGG CAT ACA GGG AGC CAG GCÀ-3′. DNA oligo probe hybridization was performed as in Blevins *et al.* (2006). The 5S rRNA 3′-flank probe was generated by T7 polymerase transcription of a PCR fragment in the presence of α[^32^P]-CTP. Primers for this template were: *T7*_5SUNIV2, 5′-*taa tac gac tca cta tag gga ga*C GAA AAG GTA TCA CAT GCC-3′ and 3′flank_F, 5′-GCA TCC CTC TTT TAT GTT TAA CCT-3′. RNA probe hybridization was performed as in Onodera *et al.* (2005) [7].

### DNA methylation assays

Genomic DNA was isolated from *Arabidopsis* inflorescences using *Nucleon PhytoPure DNA extraction kit* (Amersham). 3 µg aliquots of DNA were digested overnight with 30 U of *Alu* I, *Hpa* II or *Hae* III in the corresponding commercial buffer (New England Biolabs). Following size-separation by 0.8% agarose gel electrophoresis, gel-bound DNA was depurinated, denatured and transferred to Hybond-N+ nylon membrane (Amersham). Hybridization was performed overnight at 55°C in Church Buffer (0.25 M Sodium Phosphate, pH 7.2; 1 mM EDTA; 6.6% SDS; 1% BSA). The 5S rDNA probe was a 5S LT1 PCR fragment internally labeled with α[^32^P]-dCTP by random priming. The membrane was washed twice 5 min. with 2× SSC, 0.1% SDS and twice 20 min. with 0.1× SSC, 0.1% SDS; signal was detected by phosphoimaging. PCR analysis of cytosine methylation used 100 ng aliquots of the above digests compared to undigested DNA controls. PCR was performed for 25 cycles using Platinum Taq (Invitrogen) and primers RTPCR5S1 and 5SUNIV2 described in Vaillant *et al.* (2006) [52].

### Fluorescent *in situ* hybridization and Imaging

The 5S rDNA gene probe was labeled with biotin-16-dUTP by PCR as previously described [7]. Meristem nuclei were processed for DNA fluorescence *in situ* hybridization (FISH) as previously described [81], using 50% formamide and 2× SSC for the hybridization steps. Post-hybridization washes were performed in 50% formamide and 0.1× SSC at 42°C. Biotin-labeled probes were detected using goat anti-biotin conjugated with avidin (1∶200, Vector Laboratories) followed by streptavidin-Alexa 546 (1∶200, Molecular Probes). Nuclear DNA was counterstained with DAPI in Prolong antifade medium (Invitrogen). Preparations were inspected with a Nikon Eclipse E800i epifluorescence microscope equipped with a Photometrics Coolsnap ES Mono digital camera. Images were acquired using Phylum software, and pseudocolored and merged in Adobe Photoshop 7.

## Supporting Information

Figure S1 Evidence for 5S rRNA degradation in small RNA datasets: A) Alignment of abundant small RNA matches to 5S rRNA genes, illustrated here using a generic 5S rRNA secondary structure and based on sequencing data obtained by Rajagopalan et al. (2006) and Kasschau et al. (2007). B) Identification of likely 5S rDNA degradation products. Sequence datasets were queried for exact matches to two 16-bp interior sequences (yellow boxes) proximate to the 5′ or 3′ ends of major 5S rRNA transcripts. The most frequently obtained reads have 5′ ends that correspond to the 5S rRNA 5′ terminus, or 3′ ends that correspond to the 5S rRNA 3′ terminus.
(9.25 MB TIF)

Figure S2 Maps of 5S rDNA-derived small RNA from other tissues: A) Map of inflorescence small RNAs matching the 5S rDNA unit repeat, based on analysis of datasets from Rajagopalan et al. (2006) and Kasschau et al. (2007). Small RNA 5′-end positions are indicated on the x-axis, with sequencing reads tallied on the y-axis. Upward bars are matches to the forward strand; downward bars represent reverse strand matches. Read tallies are stacked in 10-bp bins, with size-class indicated by color. The diagram at bottom indicates the 5S rRNA gene (thick black arrow), with flanking areas being intergenic spacers. B) Same diagram as panel A, but for seedling datasets from Rajagopalan et al. (2006) and Kasschau et al. (2007). A spike in small RNAs corresponding to the 5S rRNA 5′ terminus is apparent in both inflorescence and seedling maps (short arrows), in addition to the 5S rRNA 3′ terminus spike (*) and IGS siRNA cluster (**) identified in Figure 1A.
(10.48 MB TIF)

Figure S3 DNA methylation analysis of out-crossed nrpd1 and ddm1: Southern blot comparison of (A) Hpa II and (B) Alu I-digested genomic DNA isolated from inflorescences of wild type (WT), homozygous nrpd1 (−/−), heterozygous nrpd1 (+/−), homozygous ddm1 (−/−), and heterozygous ddm1 (+/−). Hpa II tests for cytosine methylation in the symmetric CG and/or CHG contexts, while Alu I tests for asymmetric methylation. The probe corresponds to 5S LT1, as was used in Figures 2E and 3A.
(8.95 MB TIF)

Figure S4 Small RNA blot analysis of ddm1-containing double mutants: Complete siR1003 hybridization result shown only truncated in Figure 2C.
(2.85 MB TIF)

Figure S5 5S LT1 transcripts not detected in rdr6: RNA samples from inflorescences of wild type (WT), ddm1 and rdr6 were analyzed by one-step RT-PCR. Reverse transcription was performed using R primer, and PCR performed using F and R primers (Figure 1A, diagram). Control reactions were performed using ACT2 primers. RT enzyme was omitted from duplicate 5S LT1 and ACT2 reactions (no RT).
(1.85 MB TIF)

Table S1 Statistical analysis of RNA-FISH and DNA-FISH data [MS Word]: A) RNA fluorescent in situ hybridization with the siR1003 probe in wild-type (WT) and ddm1 interphase nuclei (see Figure 2B). Signals restricted to a prominent nucleolar structure devoid of DAPI staining, the nucleolar dot, were classified as “Nucleolar dot only”. Signals observed dispersed both in and outside the dot, or only outside the dot, were tallied separately. B) DNA Fluorescent in situ hybridization with the 5S rDNA probe in nuclei from WT and ddm1-containing lines (see Figure 4A). Nuclei were scored for 5S rDNA colocalization with intensely DAPI-stained chromocenters, or a lack thereof. Fisher's exact test was used to compare WT percentage localization outside chromocenters to that of ddm1, nrpd1 ddm1, rdr2 ddm1, and dcl3 ddm1 (WT→X1): all differences were significant (p<0.05). Localization outside chromocenters in ddm1 was then compared to that of nrpd1 ddm1, rdr2 ddm1, and dcl3 ddm1 (WT→X2): in this case, differences with respect to nrpd1 ddm1 and rdr2 ddm1 were significant (P<0.05).
(0.04 MB DOC)

Table S2 Small RNA distribution tables used for map generation [MS Excel]
(0.13 MB XLS)

File S1 Individual 5S rDNA sequences used for map generation [multi-FASTA]
(0.09 MB TXT)

File S2 Multiple alignment of 5S rDNA sequences [MSF alignment format]
(0.26 MB TXT)

## References

[1] Parker JS, Barford D (2006). Argonaute: A scaffold for the function of short regulatory RNAs.. Trends Biochem Sci.

[2] Meins F, Si-Ammour A, Blevins T (2005). RNA silencing systems and their relevance to plant development.. Annu Rev Cell Dev Biol.

[3] Baulcombe D (2004). RNA silencing in plants.. Nature.

[4] Pikaard CS (2006). Cell Biology of the Arabidopsis Nuclear siRNA Pathway for RNA-directed Chromatin Modification.. Cold Spring Harb Symp Quant Biol.

[5] Zhang X, Henderson IR, Lu C, Green PJ, Jacobsen SE (2007). Role of RNA polymerase IV in plant small RNA metabolism.. Proc Natl Acad Sci U S A.

[6] Herr AJ, Jensen MB, Dalmay T, Baulcombe DC (2005). RNA polymerase IV directs silencing of endogenous DNA.. Science.

[7] Onodera Y, Haag JR, Ream T, Nunes PC, Pontes O (2005). Plant nuclear RNA polymerase IV mediates siRNA and DNA methylation-dependent heterochromatin formation.. Cell.

[8] Kanno T, Huettel B, Mette MF, Aufsatz W, Jaligot E (2005). Atypical RNA polymerase subunits required for RNA-directed DNA methylation.. Nat Genet.

[9] Pontier D, Yahubyan G, Vega D, Bulski A, Saez-Vasquez J (2005). Reinforcement of silencing at transposons and highly repeated sequences requires the concerted action of two distinct RNA polymerases IV in Arabidopsis.. Genes Dev.

[10] Ream TS, Haag JR, Wierzbicki AT, Nicora CD, Norbeck A (2008). Subunit Compositions of the RNA-Silencing Enzymes Pol IV and Pol V Reveal Their Origins as Specialized Forms of RNA Polymerase II.. Mol Cell.

[11] Zhang X, Yazaki J, Sundaresan A, Cokus S, Chan SW (2006). Genome-wide high-resolution mapping and functional analysis of DNA methylation in arabidopsis.. Cell.

[12] Vaillant I, Paszkowski J (2007). Role of histone and DNA methylation in gene regulation.. Curr Opin Plant Biol.

[13] Hirochika H, Okamoto H, Kakutani T (2000). Silencing of retrotransposons in arabidopsis and reactivation by the ddm1 mutation.. Plant Cell.

[14] Lippman Z, Gendrel AV, Black M, Vaughn MW, Dedhia N (2004). Role of transposable elements in heterochromatin and epigenetic control.. Nature.

[15] Miura A, Yonebayashi S, Watanabe K, Toyama T, Shimada H (2001). Mobilization of transposons by a mutation abolishing full DNA methylation in Arabidopsis.. Nature.

[16] Hamilton A, Voinnet O, Chappell L, Baulcombe D (2002). Two classes of short interfering RNA in RNA silencing.. Embo J.

[17] Pikaard CS, Haag JR, Ream T, Wierzbicki AT (2008). Roles of RNA polymerase IV in gene silencing.. Trends Plant Sci.

[18] Xie Z, Johansen LK, Gustafson AM, Kasschau KD, Lellis AD (2004). Genetic and functional diversification of small RNA pathways in plants.. PLoS Biol.

[19] Pontes O, Li CF, Nunes PC, Haag J, Ream T (2006). The Arabidopsis chromatin-modifying nuclear siRNA pathway involves a nucleolar RNA processing center.. Cell.

[20] Zilberman D, Cao X, Jacobsen SE (2003). ARGONAUTE4 control of locus-specific siRNA accumulation and DNA and histone methylation.. Science.

[21] Qi Y, He X, Wang XJ, Kohany O, Jurka J (2006). Distinct catalytic and non-catalytic roles of ARGONAUTE4 in RNA-directed DNA methylation.. Nature.

[22] Cao X, Jacobsen SE (2002). Role of the arabidopsis DRM methyltransferases in de novo DNA methylation and gene silencing.. Curr Biol.

[23] Wierzbicki AT, Haag JR, Pikaard CS (2008). Noncoding Transcription by RNA Polymerase Pol IVb/Pol V Mediates Transcriptional Silencing of Overlapping and Adjacent Genes.. Cell.

[24] Kanno T, Mette MF, Kreil DP, Aufsatz W, Matzke M (2004). Involvement of putative SNF2 chromatin remodeling protein DRD1 in RNA-directed DNA methylation.. Curr Biol.

[25] Kanno T, Bucher E, Daxinger L, Huettel B, Bohmdorfer G (2008). A structural-maintenance-of-chromosomes hinge domain-containing protein is required for RNA-directed DNA methylation.. Nat Genet.

[26] Llave C, Kasschau KD, Rector MA, Carrington JC (2002). Endogenous and silencing-associated small RNAs in plants.. Plant Cell.

[27] Kiparisov S, Petrov A, Meskauskas A, Sergiev PV, Dontsova OA (2005). Structural and functional analysis of 5S rRNA in Saccharomyces cerevisiae.. Mol Genet Genomics.

[28] Ammons D, Rampersad J, Fox GE (1999). 5S rRNA gene deletions cause an unexpectedly high fitness loss in Escherichia coli.. Nucleic Acids Res.

[29] Szymanski M, Barciszewska MZ, Erdmann VA, Barciszewski J (2003). 5S rRNA: structure and interactions.. Biochem J.

[30] Cloix C, Tutois S, Mathieu O, Cuvillier C, Espagnol MC (2000). Analysis of 5S rDNA arrays in Arabidopsis thaliana: physical mapping and chromosome-specific polymorphisms.. Genome Res.

[31] Douet J, Tourmente S (2007). Transcription of the 5S rRNA heterochromatic genes is epigenetically controlled in Arabidopsis thaliana and Xenopus laevis.. Heredity.

[32] Cloix C, Tutois S, Yukawa Y, Mathieu O, Cuvillier C (2002). Analysis of the 5S RNA pool in Arabidopsis thaliana: RNAs are heterogeneous and only two of the genomic 5S loci produce mature 5S RNA.. Genome Res.

[33] Cloix C, Yukawa Y, Tutois S, Sugiura M, Tourmente S (2003). In vitro analysis of the sequences required for transcription of the Arabidopsis thaliana 5S rRNA genes.. Plant J.

[34] Mathieu O, Yukawa Y, Sugiura M, Picard G, Tourmente S (2002). 5S rRNA genes expression is not inhibited by DNA methylation in Arabidopsis.. Plant J.

[35] Matzke M, Kanno T, Huettel B, Jaligot E, Mette MF, Meyer P (2005). RNA-directed DNA Methylation.. Plant Epigenetics.

[36] Goll MG, Bestor TH (2005). Eukaryotic cytosine methyltransferases.. Annu Rev Biochem.

[37] Chan SW, Henderson IR, Jacobsen SE (2005). Gardening the genome: DNA methylation in Arabidopsis thaliana.. Nat Rev Genet.

[38] Finnegan EJ, Kovac KA (2000). Plant DNA methyltransferases.. Plant Mol Biol.

[39] Vongs A, Kakutani T, Martienssen RA, Richards EJ (1993). Arabidopsis thaliana DNA methylation mutants.. Science.

[40] Kankel MW, Ramsey DE, Stokes TL, Flowers SK, Haag JR (2003). Arabidopsis MET1 cytosine methyltransferase mutants.. Genetics.

[41] Saze H, Mittelsten Scheid O, Paszkowski J (2003). Maintenance of CpG methylation is essential for epigenetic inheritance during plant gametogenesis.. Nat Genet.

[42] Steimer A, Amedeo P, Afsar K, Fransz P, Mittelsten Scheid O (2000). Endogenous targets of transcriptional gene silencing in Arabidopsis.. Plant Cell.

[43] Jeddeloh JA, Stokes TL, Richards EJ (1999). Maintenance of genomic methylation requires a SWI2/SNF2-like protein.. Nat Genet.

[44] Brzeski J, Jerzmanowski A (2003). Deficient in DNA methylation 1 (DDM1) defines a novel family of chromatin-remodeling factors.. J Biol Chem.

[45] Kishimoto N, Sakai H, Jackson J, Jacobsen SE, Meyerowitz EM (2001). Site specificity of the Arabidopsis METI DNA methyltransferase demonstrated through hypermethylation of the superman locus.. Plant Mol Biol.

[46] Bartee L, Malagnac F, Bender J (2001). Arabidopsis cmt3 chromomethylase mutations block non-CG methylation and silencing of an endogenous gene.. Genes Dev.

[47] Lindroth AM, Cao X, Jackson JP, Zilberman D, McCallum CM (2001). Requirement of CHROMOMETHYLASE3 for maintenance of CpXpG methylation.. Science.

[48] Matzke M, Kanno T, Huettel B, Daxinger L, Matzke AJ (2007). Targets of RNA-directed DNA methylation.. Curr Opin Plant Biol.

[49] Teixeira FK, Heredia F, Sarazin A, Roudier F, Boccara M (2009). A Role for RNAi in the Selective Correction of DNA Methylation Defects.. Science.

[50] Kato M, Miura A, Bender J, Jacobsen SE, Kakutani T (2003). Role of CG and non-CG methylation in immobilization of transposons in Arabidopsis.. Curr Biol.

[51] Mathieu O, Jasencakova Z, Vaillant I, Gendrel AV, Colot V (2003). Changes in 5S rDNA chromatin organization and transcription during heterochromatin establishment in Arabidopsis.. Plant Cell.

[52] Vaillant I, Schubert I, Tourmente S, Mathieu O (2006). MOM1 mediates DNA-methylation-independent silencing of repetitive sequences in Arabidopsis.. EMBO Rep.

[53] Vaillant I, Tutois S, Cuvillier C, Schubert I, Tourmente S (2007). Regulation of Arabidopsis thaliana 5S rRNA Genes.. Plant Cell Physiol.

[54] Mathieu O, Reinders J, Caikovski M, Smathajitt C, Paszkowski J (2007). Transgenerational stability of the Arabidopsis epigenome is coordinated by CG methylation.. Cell.

[55] Soppe WJ, Jasencakova Z, Houben A, Kakutani T, Meister A (2002). DNA methylation controls histone H3 lysine 9 methylation and heterochromatin assembly in Arabidopsis.. Embo J.

[56] Douet J, Blanchard B, Cuvillier C, Tourmente S (2008). Interplay of RNA Pol IV and ROS1 during postembryonic 5S rDNA chromatin remodelling.. Plant Cell Physiol.

[57] Kasschau KD, Fahlgren N, Chapman EJ, Sullivan CM, Cumbie JS (2007). Genome-Wide Profiling and Analysis of Arabidopsis siRNAs.. PLoS Biol.

[58] Creusot F, Fouilloux E, Dron M, Lafleuriel J, Picard G (1995). The CIC library: a large insert YAC library for genome mapping in Arabidopsis thaliana.. Plant J.

[59] Rajagopalan R, Vaucheret H, Trejo J, Bartel DP (2006). A diverse and evolutionarily fluid set of microRNAs in Arabidopsis thaliana.. Genes Dev.

[60] Vazquez F, Vaucheret H, Rajagopalan R, Lepers C, Gasciolli V (2004). Endogenous trans-acting siRNAs regulate the accumulation of Arabidopsis mRNAs.. Mol Cell.

[61] Deleris A, Gallego-Bartolome J, Bao J, Kasschau KD, Carrington JC (2006). Hierarchical Action and Inhibition of Plant Dicer-Like Proteins in Antiviral Defense.. Science.

[62] Blevins T, Rajeswaran R, Shivaprasad PV, Beknazariants D, Si-Ammour A (2006). Four plant Dicers mediate viral small RNA biogenesis and DNA virus induced silencing.. Nucleic Acids Res.

[63] Henderson IR, Zhang X, Lu C, Johnson L, Meyers BC (2006). Dissecting Arabidopsis thaliana DICER function in small RNA processing, gene silencing and DNA methylation patterning.. Nat Genet.

[64] Gasciolli V, Mallory AC, Bartel DP, Vaucheret H (2005). Partially redundant functions of Arabidopsis DICER-like enzymes and a role for DCL4 in producing trans-acting siRNAs.. Curr Biol.

[65] Lippman Z, May B, Yordan C, Singer T, Martienssen R (2003). Distinct mechanisms determine transposon inheritance and methylation via small interfering RNA and histone modification.. PLoS Biol.

[66] Kakutani T, Munakata K, Richards EJ, Hirochika H (1999). Meiotically and mitotically stable inheritance of DNA hypomethylation induced by ddm1 mutation of Arabidopsis thaliana.. Genetics.

[67] Cao X, Aufsatz W, Zilberman D, Mette MF, Huang MS (2003). Role of the DRM and CMT3 methyltransferases in RNA-directed DNA methylation.. Curr Biol.

[68] Heslop-Harrison JS (2000). Comparative genome organization in plants: from sequence and markers to chromatin and chromosomes.. Plant Cell.

[69] Pikaard CS (1999). Nucleolar dominance and silencing of transcription.. Trends Plant Sci.

[70] Plohl M, Luchetti A, Mestrovic N, Mantovani B (2008). Satellite DNAs between selfishness and functionality: structure, genomics and evolution of tandem repeats in centromeric (hetero)chromatin.. Gene.

[71] Woo HR, Dittmer TA, Richards EJ (2008). Three SRA-domain methylcytosine-binding proteins cooperate to maintain global CpG methylation and epigenetic silencing in Arabidopsis.. PLoS Genet.

[72] Slotkin RK, Vaughn M, Borges F, Tanurdzic M, Becker JD (2009). Epigenetic reprogramming and small RNA silencing of transposable elements in pollen.. Cell.

[73] Chan SW, Henderson IR, Zhang X, Shah G, Chien JS (2006). RNAi, DRD1, and histone methylation actively target developmentally important non-CG DNA methylation in arabidopsis.. PLoS Genet.

[74] Allen E, Xie Z, Gustafson AM, Carrington JC (2005). microRNA-directed phasing during trans-acting siRNA biogenesis in plants.. Cell.

[75] Akbergenov R, Si-Ammour A, Blevins T, Amin I, Kutter C (2006). Molecular characterization of geminivirus-derived small RNAs in different plant species.. Nucleic Acids Res.

[76] Xie Z, Allen E, Wilken A, Carrington JC (2005). DICER-LIKE 4 functions in trans-acting small interfering RNA biogenesis and vegetative phase change in Arabidopsis thaliana.. Proc Natl Acad Sci U S A.

[77] Daxinger L, Hunter B, Sheikh M, Jauvion V, Gasciolli V (2008). Unexpected silencing effects from T-DNA tags in Arabidopsis.. Trends Plant Sci.

[78] Jacobsen SE, Running MP, Meyerowitz EM (1999). Disruption of an RNA helicase/RNAse III gene in Arabidopsis causes unregulated cell division in floral meristems.. Development.

[79] Ouyang S, Buell CR (2004). The TIGR Plant Repeat Databases: a collective resource for the identification of repetitive sequences in plants.. Nucleic Acids Res.

[80] Thompson JD, Higgins DG, Gibson TJ (1994). CLUSTAL W: improving the sensitivity of progressive multiple sequence alignment through sequence weighting, position-specific gap penalties and weight matrix choice.. Nucleic Acids Res.

[81] Pontes O, Lawrence RJ, Neves N, Silva M, Lee JH (2003). Natural variation in nucleolar dominance reveals the relationship between nucleolus organizer chromatin topology and rRNA gene transcription in Arabidopsis.. Proc Natl Acad Sci U S A.

