# Supplementary figures and images for: Heterochromatic siRNAs and DDM1 Independently Silence Aberrant 5S rDNA Transcripts in Arabidopsis
Source: PLoS One. 2009 Jun 16;4(6):e5932. doi: 10.1371/journal.pone.0005932 (PMC2691480; doi:10.1371/journal.pone.0005932)

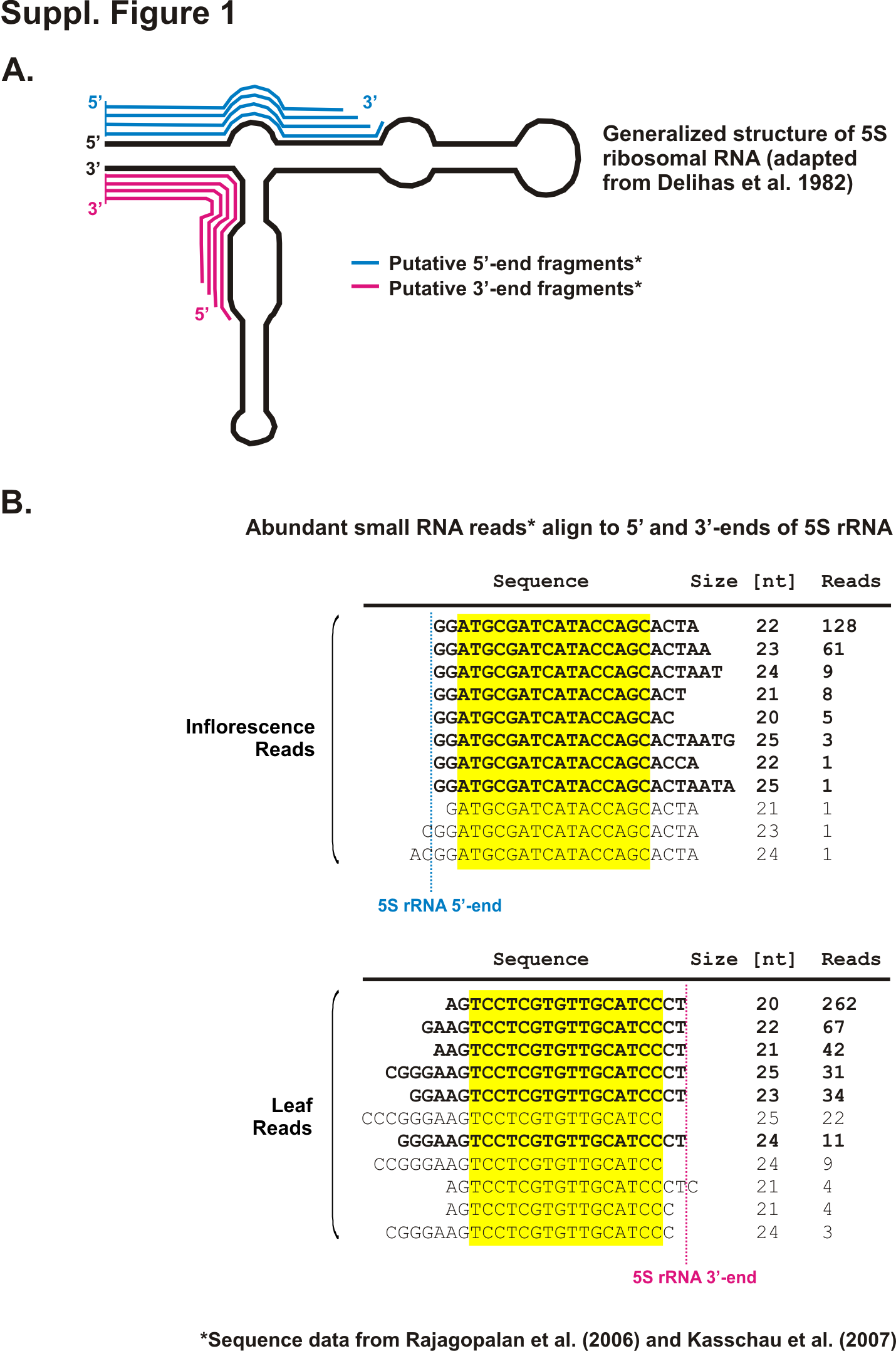

Supplement: Figure S1 — Evidence for 5S rRNA degradation in small RNA datasets: A) Alignment of abundant small RNA matches to 5S rRNA genes, illustrated here using a generic 5S rRNA secondary structure and based on sequencing data obtained by Rajagopalan et al. (2006) and Kasschau et al. (2007). B) Identification of likely 5S rDNA degradation products. Sequence datasets were queried for exact matches to two 16-bp interior sequences (yellow boxes) proximate to the 5′ or 3′ ends of major 5S rRNA transcripts. The most frequently obtained reads have 5′ ends that correspond to the 5S rRNA 5′ terminus, or 3′ ends that correspond to the 5S rRNA 3′ terminus. (9.25 MB TIF) [file pone.0005932.s001.tif]

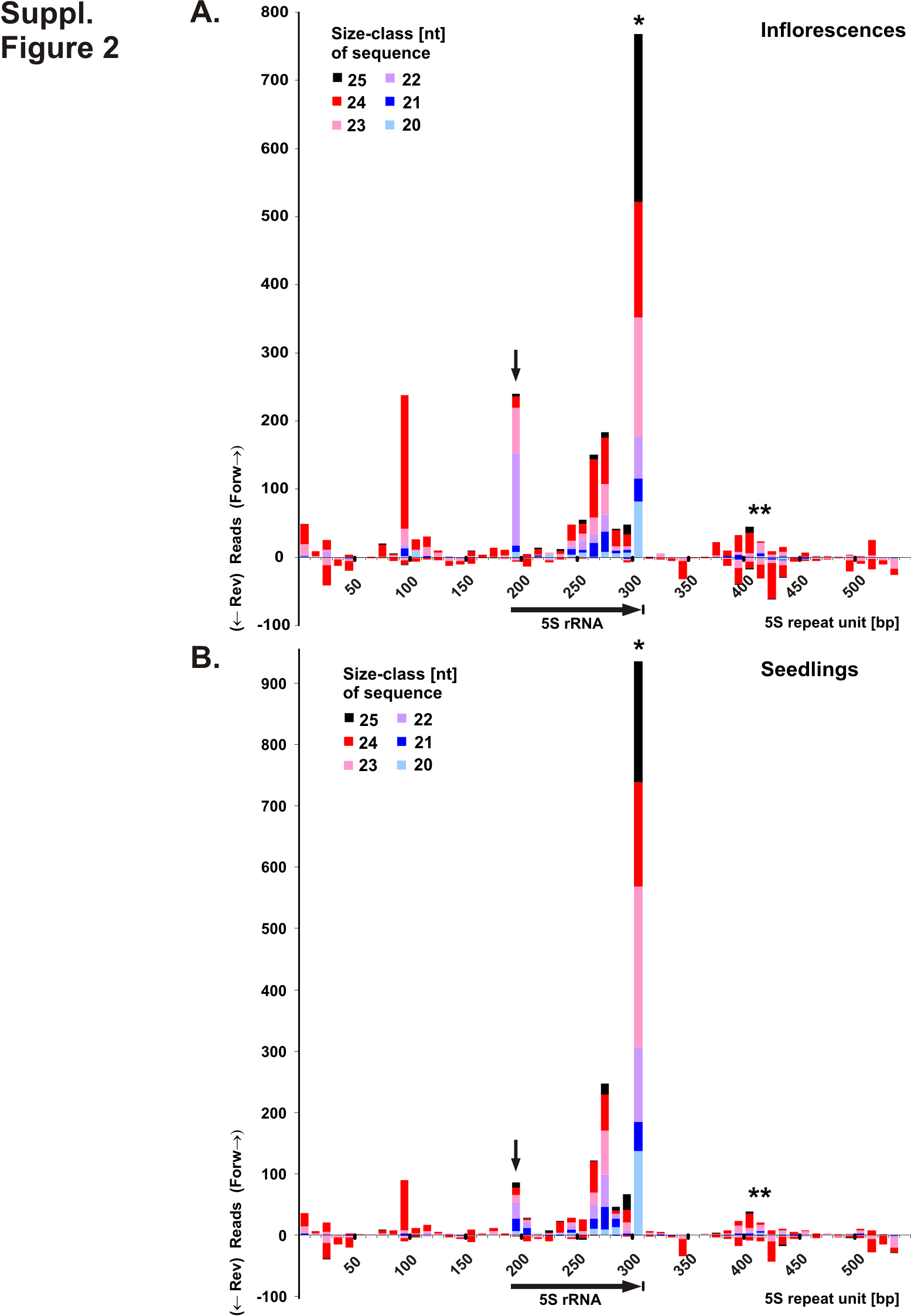

Supplement: Figure S2 — Maps of 5S rDNA-derived small RNA from other tissues: A) Map of inflorescence small RNAs matching the 5S rDNA unit repeat, based on analysis of datasets from Rajagopalan et al. (2006) and Kasschau et al. (2007). Small RNA 5′-end positions are indicated on the x-axis, with sequencing reads tallied on the y-axis. Upward bars are matches to the forward strand; downward bars represent reverse strand matches. Read tallies are stacked in 10-bp bins, with size-class indicated by color. The diagram at bottom indicates the 5S rRNA gene (thick black arrow), with flanking areas being intergenic spacers. B) Same diagram as panel A, but for seedling datasets from Rajagopalan et al. (2006) and Kasschau et al. (2007). A spike in small RNAs corresponding to the 5S rRNA 5′ terminus is apparent in both inflorescence and seedling maps (short arrows), in addition to the 5S rRNA 3′ terminus spike (*) and IGS siRNA cluster (**) identified in Figure 1A. (10.48 MB TIF) [file pone.0005932.s002.tif]

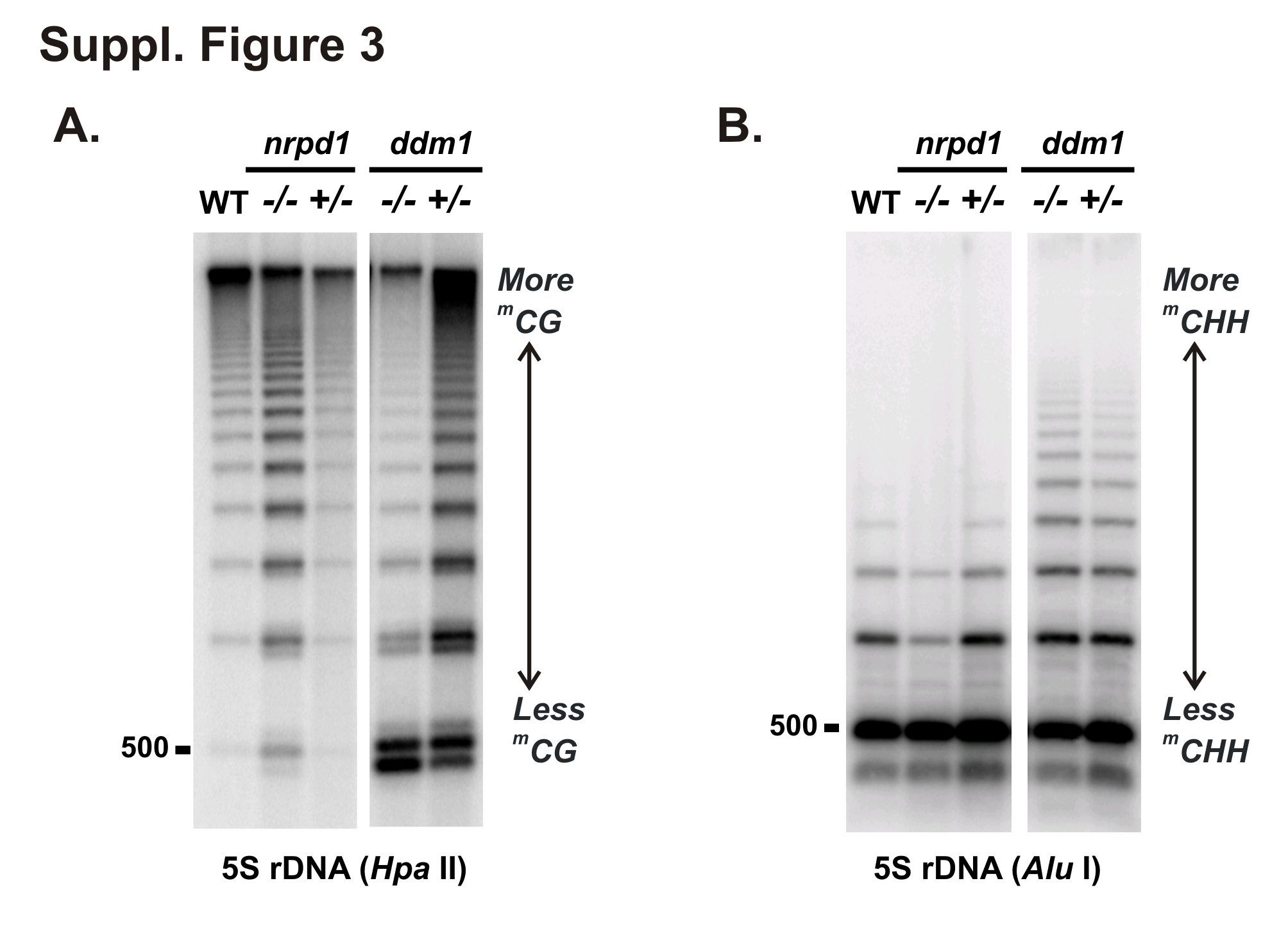

Supplement: Figure S3 — DNA methylation analysis of out-crossed nrpd1 and ddm1: Southern blot comparison of (A) Hpa II and (B) Alu I-digested genomic DNA isolated from inflorescences of wild type (WT), homozygous nrpd1 (−/−), heterozygous nrpd1 (+/−), homozygous ddm1 (−/−), and heterozygous ddm1 (+/−). Hpa II tests for cytosine methylation in the symmetric CG and/or CHG contexts, while Alu I tests for asymmetric methylation. The probe corresponds to 5S LT1, as was used in Figures 2E and 3A. (8.95 MB TIF) [file pone.0005932.s003.tif]

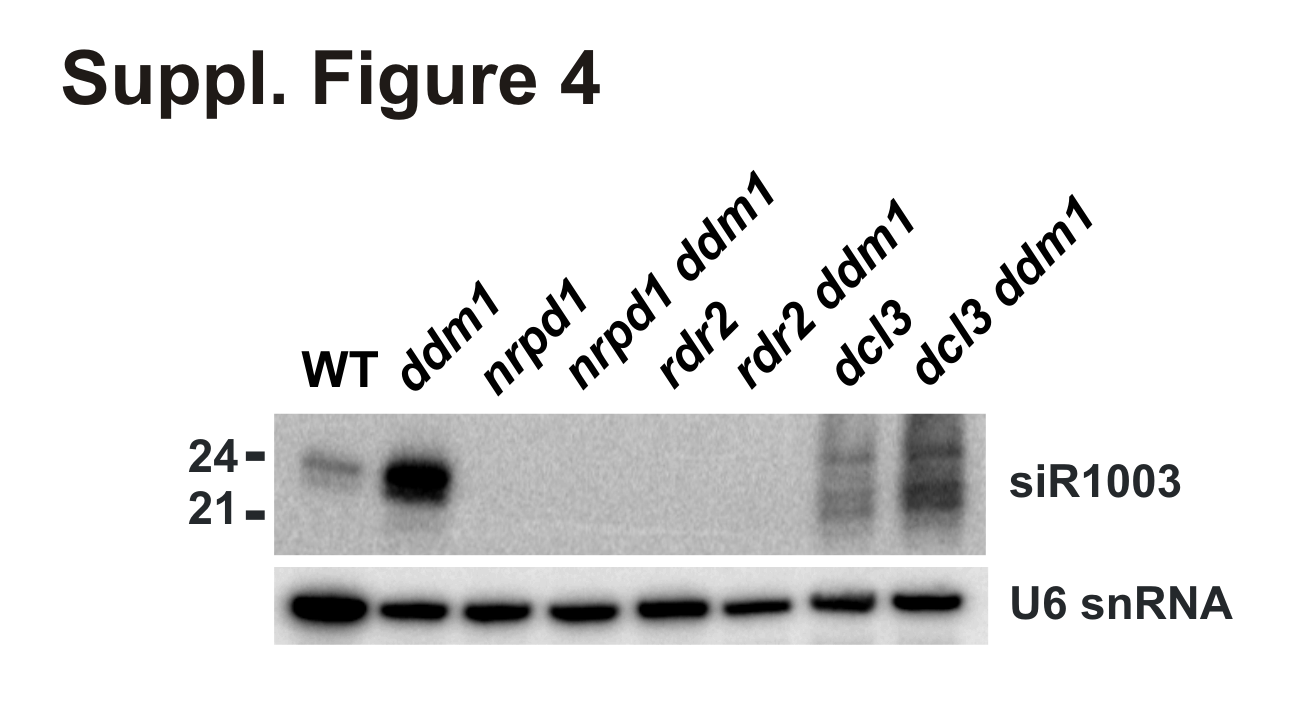

Supplement: Figure S4 — Small RNA blot analysis of ddm1-containing double mutants: Complete siR1003 hybridization result shown only truncated in Figure 2C. (2.85 MB TIF) [file pone.0005932.s004.tif]

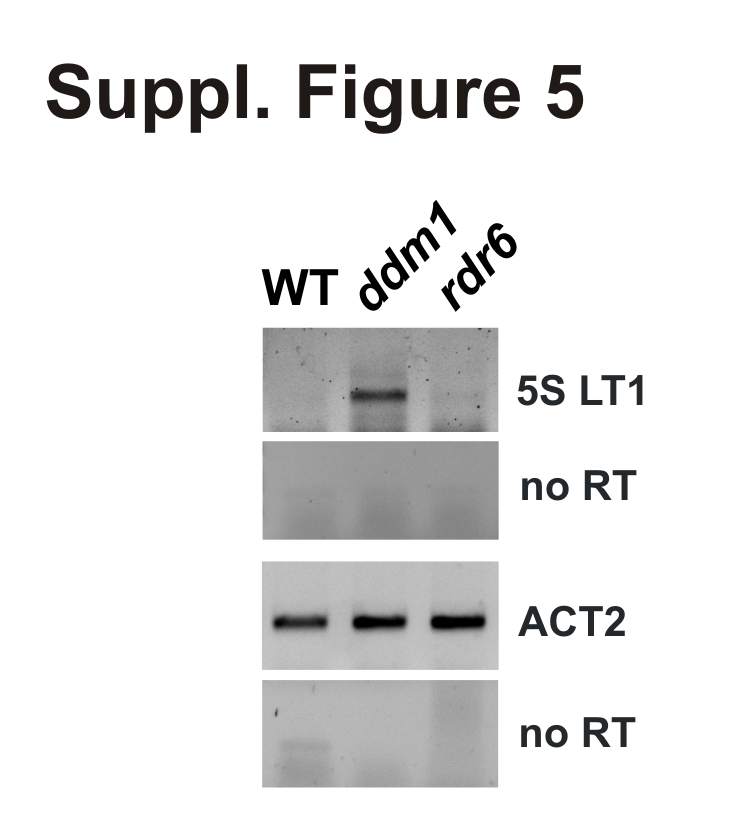

Supplement: Figure S5 — 5S LT1 transcripts not detected in rdr6: RNA samples from inflorescences of wild type (WT), ddm1 and rdr6 were analyzed by one-step RT-PCR. Reverse transcription was performed using R primer, and PCR performed using F and R primers (Figure 1A, diagram). Control reactions were performed using ACT2 primers. RT enzyme was omitted from duplicate 5S LT1 and ACT2 reactions (no RT). (1.85 MB TIF) [file pone.0005932.s005.tif]
