# Supplementary material for: Heterochromatic siRNAs and DDM1 Independently Silence Aberrant 5S rDNA Transcripts in Arabidopsis
Source: PLoS One. 2009 Jun 16;4(6):e5932. doi: 10.1371/journal.pone.0005932 (PMC2691480; doi:10.1371/journal.pone.0005932)
Supplement: Table S1 — Statistical analysis of RNA-FISH and DNA-FISH data [MS Word]: A) RNA fluorescent in situ hybridization with the siR1003 probe in wild-type (WT) and ddm1 interphase nuclei (see Figure 2B). Signals restricted to a prominent nucleolar structure devoid of DAPI staining, the nucleolar dot, were classified as “Nucleolar dot only”. Signals observed dispersed both in and outside the dot, or only outside the dot, were tallied separately. B) DNA Fluorescent in situ hybridization with the 5S rDNA probe in nuclei from WT and ddm1-containing lines (see Figure 4A). Nuclei were scored for 5S rDNA colocalization with intensely DAPI-stained chromocenters, or a lack thereof. Fisher's exact test was used to compare WT percentage localization outside chromocenters to that of ddm1, nrpd1 ddm1, rdr2 ddm1, and dcl3 ddm1 (WT→X1): all differences were significant (p<0.05). Localization outside chromocenters in ddm1 was then compared to that of nrpd1 ddm1, rdr2 ddm1, and dcl3 ddm1 (WT→X2): in this case, differences with respect to nrpd1 ddm1 and rdr2 ddm1 were significant (P<0.05). (0.04 MB DOC) [file pone.0005932.s006.doc]

**Table S1. Statistical analysis of RNA-FISH and DNA-FISH data**

| **Part A.** | **RNA-FISH analysis: localization pattern, siR1003 probe** | | | |
| --- | --- | --- | --- | --- |
| **Genotype** | Nucleolar dot only | Nucleolar dot and dispersed signals in the nucleoplasm | Dispersed signals in the nucleolus and nucleoplasm | cells analyzed  (n) |
| **WT** | 83% | 10% | 7% | 136 |
| ***ddm1*** | 6% | 26% | 68% | 158 |

| **Part B.** | **DNA-FISH analysis: localization pattern, 5S rDNA probe** | | | | |
| --- | --- | --- | --- | --- | --- |
| **Genotype** | Colocalized w/ chromocenters | Not colocalized w/ chromocenters | Cells (n) analyzed | **Fisher’s exact test** | |
| **WT** | 77% | **23%** | 134 | **WT 🡪 *x_1_*** |  |
| ***ddm1*** | 48% | **52%** | 158 | * P<0.0001 | ***ddm1* 🡪 *x_2_*** |
| ***nrpd1 ddm1*** | 32% | **68%** | 186 | * P<0.0001 | * P = 0.0301 |
| ***rdr2 ddm1*** | 27% | **73%** | 215 | * P<0.0001 | * P = 0.0034 |
| ***dcl3 ddm1*** | 42% | **58%** | 190 | * P<0.0001 | P = 0.4774 |

**A)** RNA fluorescent *in situ* hybridization with the siR1003 probe in wild-type (WT) and *ddm1* interphase nuclei (see **Figure 2B**). Signals restricted to a prominent nucleolar structure devoid of DAPI staining, the nucleolar dot, were classified as “Nucleolar dot only”. Signals observed dispersed both in and outside the dot, or only outside the dot, were tallied separately. **B)** DNA Fluorescent *in situ* hybridization with the 5S rDNA probe in nuclei from WT and *ddm1*-containing lines (see **Figure 4A**). Nuclei were scored for 5S rDNA colocalization with intensely DAPI-stained chromocenters, or a lack thereof. Fisher’s exact test was used to compare WT percentage localization outside chromocenters to that of *ddm1*, *nrpd1 ddm1*, *rdr2 ddm1*, and *dcl3 ddm1* (WT 🡪 *x_1_*): all differences were significant (p < 0.05). Localization outside chromocenters in *ddm1* was then compared to that of *nrpd1 ddm1*, *rdr2 ddm1*, and *dcl3 ddm1* (WT 🡪 *x_2_*): in this case, differences with respect to *nrpd1 ddm1* and *rdr2 ddm1* were significant (P < 0.05).
